# Mechanical support for high-risk coronary artery bypass grafting

**DOI:** 10.1007/s12055-018-0740-1

**Published:** 2018-10-23

**Authors:** Jason M. Ali, Yasir Abu-Omar

**Affiliations:** Department of Cardiothoracic Surgery, Royal Papworth Hospital, Papworth Everard, Cambridge, CB23 3RE UK

**Keywords:** Mechanical circulatory support, Intra-aortic balloon pump, ECMO

## Abstract

Mechanical circulatory support devices are being used to an increasing extent. The use of these devices as an adjunct to cardiac surgery to support ventricular function has contributed to improved outcomes for the highest risk patients. In the context of patients undergoing coronary artery bypass grafting, there are several potential indications for mechanical circulatory support: preoperatively in the setting of acute cardiogenic shock, or in patients with intractable angina with or without haemodynamic compromise; at induction of anaesthesia prophylactically in patients with critical coronary anatomy and/or severely impaired left ventricular function; intraoperatively in the setting of failure to wean from cardiopulmonary bypass; or postoperatively in patients who develop an intractable low cardiac output state. The use of the intra-aortic balloon pump, veno-arterial extracorporeal membrane oxygenation, TandemHeart, Impella and central ventricular assist devices will be considered in the setting of high-risk patients undergoing coronary artery bypass grafting.

## Introduction

There has been a significant increase in the utilisation of mechanical circulatory support (MCS) technology in cardiovascular medicine and surgery over recent years [1]. Historically, MCS was reserved primarily for patients with severe cardiovascular dysfunction who were deemed candidates for transplantation, with MCS used as a bridge to transplantation. Nowadays, advanced MCS devices are commonplace in the cardiothoracic intensive care unit and the indications for their use have broadened significantly to the extent that these devices may now be even used prophylactically. The use of these devices as an adjunct to cardiac surgery to support ventricular function has contributed to improved outcomes for the highest risk patients [1, 2].

## High-risk coronary artery bypass grafting

Despite advances and developments within interventional cardiology, coronary artery bypass grafting (CABG) remains the guideline treatment of choice for patients with severe coronary artery disease due to the excellent outcomes achieved [2]. Patients with chronic heart failure or acute cardiogenic shock, who have the highest perioperative risk, represent the most likely group to benefit from the use of MCS in the perioperative period.

### Chronic heart failure

Patients with chronic heart failure and left ventricular dysfunction secondary to ischaemic heart disease represent a high-risk surgical group. The benefit of surgery in this cohort of patients was demonstrated in the Surgical Treatment for Ischemic Heart Failure (STICH) trial [3]. This randomised controlled trial compared CABG with the best medical therapy in 1212 patients with ejection fraction (EF) < 35%. Although the primary outcome, all-cause mortality, was not significantly different between the groups, there was a reduced mortality rate from a cardiovascular cause (hazard ratio (HR) 0.81; 95% CI 0.66 to 1.00; *p* = 0.05) and all-cause mortality or hospitalisation for heart failure (HR 0.84; 95% CI 0.71–0.98; *p* = 0.03). It was notable that by the end of their follow-up period, 17% of the medical group had undergone CABG. A large propensity-matched study from the USA similarly comparing CABG with the best medical therapy observed similar results and demonstrated a survival advantage to 10 years postoperatively [4]. Following from these observations, CABG is a class I recommendation for patients with severe coronary artery disease and chronic heart failure with left ventricular dysfunction (EF < 35%) [2].

### Acute cardiogenic shock

Cardiogenic shock is the primary cause of in-hospital mortality following an acute myocardial infarction (MI), with an incidence of 6–8% and mortality as high as 40% [5]. Cardiogenic shock complicating MI is caused by left ventricular failure in approximately 80% of cases. Mechanical complications, such as papillary muscle rupture with severe mitral valve incompetence, ventricular septal defect or free wall rupture, account for the remaining 20%. The benefit of early revascularisation was demonstrated in the ‘Should We Emergently Revascularize Occluded Coronaries for Cardiogenic Shock’ (SHOCK) randomised controlled trial [6]. Patients with cardiogenic shock due to acute MI undergoing emergency revascularisation with percutaneous coronary intervention (PCI) or CABG had improved long-term survival when compared with those of initial intensive medical therapy. All-cause mortality at 6 months was lower in the group assigned to revascularisation (50.3% vs. 63.1%, respectively; RR 0.80; 95% CI 0.65–0.98; *p* = 0.03). As such, CABG is a class I recommendation for patients with acute cardiogenic shock and coronary anatomy not suitable for PCI [2].

### Postoperative outcomes

Despite being the guideline treatment of choice, CABG in patients with severe left ventricular dysfunction, due either to chronic heart failure, or cardiogenic shock following an acute MI, is associated with high surgical risk. Studies report in-hospital mortality for patients with severely impaired left ventricular function between 4.6 and 11% which is significantly higher than that observed for patients with good ventricular function, where in-hospital mortality rates in the range 1–3% are common [7, 8]. For patients with acute cardiogenic shock post-MI, the mortality rate following surgery is even higher. A recent large national registry analysis in the USA revealed a postoperative mortality rate of 18.7% in this patient cohort [9]. Reassuringly, they observed that mortality rate is decreasing with time. Over their period of study, there was a significant increase in use of MCS in this patient cohort, leading them to suggest that increased utilisation of MCS has contributed to improving outcomes in this high-risk patient cohort.

### Use of MCS in patients undergoing CABG

MCS can be instituted at different time points in the perioperative pathway depending on the degree of impaired ventricular function and symptom status of the patient (Table 1).

**Table 1:** Indications for mechanical circulatory support in patients undergoing CABG

Indications for MCS in patients undergoing CABG	
Preoperatively	
Acute cardiogenic shock—such as after an acute myocardial infarction	
Symptomatic angina despite maximal medical therapy	
Prophylactically in patients with critical coronary anatomy ± haemodynamic compromise	
At induction of anaesthesia	
Prophylactically in patients with severe ventricular impairment	
Intraoperatively	
Failure to wean from cardiopulmonary bypass	
Postoperatively	
Development of low cardiac output state in the intensive care unit	

There are several forms of MCS that can be utilised, and these will be considered in turn. They include intra-aortic balloon pump (IABP), extracorporeal membrane oxygenation (ECMO) and percutaneous or surgical ventricular assist devices (VAD) (Table 2) [10].

**Table 2:** Comparison of mechanical circulatory support devices, modified from Werdan et al. [10]

	Intra-aortic balloon pump	Veno-arterial ECMO	TandemHeart	Impella	Central ventricular assist device
Pump mechanism	Pneumatic	Centrifugal	Axial	Axial	Centrifugal
Insertion technique	Descending aorta via femoral artery	Peripheral (e.g. femoral artery and vein cannulation) or central (right atrial and ascending aorta cannulation)	Cannula into left atrium. Inserted through femoral vein with trans-septal puncture	Into left ventricle retrogradely via femoral artery and through aortic valve	Central cannulation (left atrial and ascending aorta)
Risk of limb ischaemia	+	+++ (if peripheral)	++	++	−
Anticoagulation	+	+++	++	++	+++
Haemolysis	+	++	++	++	++
Haemodynamic support					
Cardiac output increase	0.5–1 L/min	> 4.5 L/min	4 L/min	2.5 or 5.0 L/min	> 4.5 L/min
Afterload	Reduced	Increased	Increased	Unchanged	Increased
LV preload	Slightly reduced	Reduced	Reduced	Slightly reduced	Reduced
LV stroke volume	Slight increase	Reduced	Reduced	Reduced	Reduced
Coronary perfusion	Slight increase	Unknown	Unknown	Unknown	Unknown
Systemic perfusion	Little change	Improved	Improved	Improved	Improved

*ECMO* extracorporeal membrane oxygenator, *LV* left ventricle

## IABP

The IABP is the commonest form of mechanical support utilised in cardiothoracic practice. Its first use was in the 1960s following the work of Kantrowitz who identified that ‘diastolic augmentation’ could be utilised to improve myocardial oxygenation [11]. The IABP comprises a drive console with a pump and a double-lumen balloon catheter that is typically introduced percutaneously into the femoral artery using the Seldinger technique. One lumen is used for pressure monitoring, and the other is connected to the pump through which helium is delivered and removed into the balloon. Helium is used due to its low viscosity (permitting rapid transfer into the balloon) and high blood solubility (reducing the impact of gas embolism should the balloon rupture). The tip of the balloon is positioned just distal to the left subclavian artery to reduce the risk of the balloon occluding the cerebral arteries proximally and abdominal visceral arteries distally. The position can be confirmed during placement with fluoroscopy or transoesophageal echocardiography, or on a subsequent chest radiograph. Patients should receive therapeutic anticoagulation, if no contraindication, to reduce the risk of thromboembolic events.

### Counterpulsation

The haemodynamic effects of the IABP depend upon the counterpulsation that results from balloon inflation and deflation at precise points in the cardiac cycle which are controlled by the drive console, using either pressure or electrocardiogram triggers. The aim is to improve ventricular performance by increasing myocardial oxygen supply whilst simultaneously reducing myocardial oxygen demand. Balloon inflation is timed to occur immediately after aortic valve closure resulting in augmentation of the diastolic pressure (‘diastolic augmentation’). This results in increased coronary artery perfusion and therefore myocardial oxygen delivery. Balloon deflation is timed to occur immediately prior to opening of the aortic valve, creating a vacuum effect leading to reduced afterload which leads to reduced myocardial oxygen demand. The reduced afterload can additionally result in improved contractility and increased cardiac output, although the increase in cardiac output is believed to be small (0.5–1 L/min at most), and of course dependent upon the ventricular function [12].

The degree of haemodynamic support can be modulated by altering the inflation volume of the balloon, or the proportion of beats that are augmented. These strategies are used to facilitate weaning of IABP support.

### Indications

The IABP is the most readily available form of mechanical support and as such the indications for use are wide in the context of patients undergoing CABG:

- Cardiogenic shock—IABP utilised preoperatively to improve the patients haemodynamic status in shock refractory to pharmacological management, as a bridge to revascularisation
- Unstable angina—IABP utilised preoperatively in patients with symptoms and signs of myocardial ischaemia despite optimal medical therapy, leading to improved myocardial perfusion and therefore improved haemodynamic status
- Prophylactically—IABP utilised perioperatively to optimise patient haemodynamics and minimise the risk of perioperative myocardial ischaemia in patients with critical coronary anatomy or in patients with poor left ventricular function in whom it is anticipated there is high risk of experiencing problems weaning from cardiopulmonary bypass (CPB)
- Facilitate weaning from CPB—IABP utilised to facilitate weaning from CPB in the setting of low cardiac output refractory to pharmacological support
- Postoperative low cardiac output syndrome—IABP utilised postoperatively in patients developing low cardiac output in the ITU, refractory to pharmacological therapy

### Contraindications

The principal contraindications of IABP use include significant aortic regurgitation (since diastolic augmentation in part depends upon a competent valve, and regurgitation will be made worse) and severe peripheral arterial or aortic disease such as atherosclerosis or aneurysms since this increases the risk of vascular complications. Others include aortic dissection, uncontrolled sepsis and uncontrolled bleeding disorders.

### Complications

A large registry study reported that the incidence of complications in patients with an IABP is 7%, with major complications occurring in 2.6%. IABP-related mortality was estimated to be 0.5% [13]. The commonest complications are vascular and include limb ischaemia and vascular trauma (dissection or laceration) leading to false aneurysm, haematoma or haemorrhage. Distal pulses should be regularly examined and surveillance for the development of compartment syndrome. Incorrectly positioned or incorrectly sized IABP catheters can lead to compromised abdominal visceral perfusion. Non-vascular complications include cerebrovascular accident, thrombocytopaenia from platelet deposition on the balloon and due to mechanical disruption, haemolysis, infection and complications of immobility in cases of prolonged therapy due to the requirement to be bed-bound. Balloon rupture is a rare but serious complication that can result in gas embolism and subsequent thrombus deposition on the static balloon with subsequent embolism.

### Clinical outcomes

The use of IABP preoperatively has been the subject of much controversy within the literature since publication of the findings of the Intra-aortic Balloon Pump in Cardiogenic Shock (IABP-SHOCK II) trial [14]. This randomised controlled trial included 600 patients with cardiogenic shock complicating acute MI, who were assigned to IABP or no IABP. The primary endpoint of 30-day mortality was not reduced with the use of IABP (39.7% IABP vs. 41.3% control; RR 0.96; 95% CI 0.79–1.17; *p* = 0.69) and there was no long-term benefit. This resulted in downgrading the recommendation to use IABP in the latest guidelines. The routine use of IABP for cardiogenic shock following MI is now a class III recommendation and use for haemodynamic instability/cardiogenic shock due to mechanical complications is downgraded to a class IIa recommendation [2]. It is worth highlighting that only 5% of the patients included underwent revascularisation with CABG, and so these results may not be entirely generalisable to the surgical population.

Nevertheless, there remains ongoing debate as to the clinical utility of prophylactic IABP use preoperatively in patients undergoing CABG. There are several large systematic reviews and meta-analyses that support the use of prophylactic IABP. Wang et al. demonstrated that preoperative IABP use resulted in significantly improved short-term mortality (OR 0.26; 95% CI 0.13–0.53; *p* < 0.001) and significantly reduced the incidence of acute kidney injury in high-risk patients [15]. Poirier et al. demonstrated in their meta-analysis that there was a significant reduction in mortality (OR 0.43; 95% CI 0.25–0.76; *p* = 0.003), length of ITU stay and length of hospital stay associated with the use of preoperative IABP [16]. They observed an incidence of complications associated with the IABP of 3%. Deppe et al. similarly demonstrated benefits associated with preoperative IABP use, with a significant reduction in hospital mortality (OR 0.44; 95% CI 0.24–0.77; *p* = 0.003), incidence of MI, low cardiac output state, renal dysfunction and cerebrovascular accident [17]. Most recently, Rampersad et al. have reaffirmed these observations, including recent studies in their meta-analysis [18]. They concluded that use of preoperative IABP is associated with a significant mortality benefit (OR 0.48; 95% CI 0.30–0.76; *p* = 0.002), and a significant reduction in major adverse cardiovascular and cerebrovascular events. Despite this array of data, there are some limitations of these meta-analyses such as the fact that several of the randomised controlled trials (RCT) included are from a single centre. There is also some evidence that refutes these claims. For example, a large multicentre observational study by Baskett et al. concluded that no benefit was derived from preoperative IABP use, and in fact, there was an increased mortality in the IABP group [19]. Two further recent studies—one an RCT—have also demonstrated no difference between clinical endpoints in patients receiving IABP therapy prophylactically. In fact, they demonstrate increased ITU length of stay and duration of inotrope use in the IABP groups [20, 21].

Putting this data together, it seems likely that there is some benefit to preoperative use of IABP in some high-risk patients. However, a large well-designed RCT is required to reconcile the previous results and clarify the characteristics of the patients who will benefit the most from this therapy.

## Veno-arterial ECMO

ECMO is an advanced form of temporary life support that can be utilised to aid respiratory and/or cardiac function which has been in use since the 1970s. It has evolved from CPB technology and similarly involves draining venous blood through an extracorporeal circuit, in which gas exchange occurs through a membrane oxygenator, and return of the blood to the body. When required to provide circulatory support, the oxygenated blood is reinfused into the systemic arterial circulation (hence veno-arterial ECMO (VA-ECMO)). ECMO can also be utilised to support respiratory function alone, and in this setting, the oxygenated blood is returned to the venous circulation (veno-venous ECMO), relying on intrinsic cardiac function for cardiac output [22].

### Circuit design

Many circuit configurations can be constructed for VA-ECMO. The common components include a venous cannula which drains deoxygenated blood through heparin-bonded tubing to a membrane oxygenator and then to a non-pulsatile centrifugal pump through to an arterial cannula. VA-ECMO can be instituted at the patients’ bedside with percutaneous cannulation, or in theatre centrally with similar cannulation to that used for CPB. When performed percutaneously, a typical configuration will use femoral venous and arterial cannulation. Cannulation may be performed using the Seldinger technique with ultrasound guidance, or with a surgical cut-down. The patient must be therapeutically anticoagulated to minimise the risk of thrombosis.

During VA-ECMO, it is important that cardiac contractility is maintained to avoid left ventricular stasis and distension, pulmonary hypertension or intracardiac thrombus formation. This can be achieved with concomitant inotrope and IABP use. Persistent ventricular distension and pulmonary hypertension will need to be managed with left ventricle (LV) venting [23].

### Indications

The use of VA-ECMO and other advanced MCS devices is generally indicated in patients with refractory cardiogenic shock despite maximal inotropic support and use of an IABP [24]. The goal is to prevent development of end-organ injury, whilst facilitating myocardial recovery. In the context of patients undergoing CABG, the indications for ECMO include the following:

- Refractory cardiogenic shock following acute MI as a bridge to revascularisation preoperatively
- Post-cardiotomy cardiogenic shock with failure to wean from CPB—despite maximal pharmacological support and use of IABP
- Postoperative cardiogenic shock refractory to pharmacological therapy and use of an IABP

### Contraindications

The Extracorporeal Life Support Organization (ELSO) maintains a registry of all patients treated with extracorporeal life support and publishes recommendations for the use of ECMO [25]. VA-ECMO is not a long-term therapy and should only be used in patients in whom there is anticipated early recovery or is being used as a bridge to more definitive management, for example transplantation or long-term VAD support. Therefore, ECMO is contraindicated in patients with non-recoverable cardiac failure who are not candidates for transplantation or VAD implantation.

### Complications

ECMO has significant associated morbidity. The major complications are thrombotic and haemorrhagic highlighting the importance of ensuring adequate but not excessive anticoagulation. Patients on ECMO are bed-bound similar to those with IABP and so suffer associated complications. A recent meta-analysis has summarised the complications of VA-ECMO (Table 3) [26].

**Table 3:** Complications of extracorporeal membrane oxygenation (ECMO)

Complications of ECMO	Incidence (%)
Acute kidney injury	56
Renal replacement therapy	46
Re-thoracotomy for bleeding or tamponade in post-cardiotomy patients	42
Major bleeding	41
Significant infection	30
Lower extremity ischaemia	17
Neurological complications	14
Compartment syndrome/fasciotomy	10
Lower extremity amputation	5

### Clinical outcomes

Survival of patients supported with VA-ECMO is dependent on a range of factors including the underlying diagnosis and the presence of end-organ injury prior to commencement. The ELSO registry data reports that 62% of patients treated for all indications with VA-ECMO survive extracorporeal life support, with 45% surviving to hospital discharge or transfer [26].

In the context of CABG, the commonest scenario for the requirement of VA-ECMO is development of post-cardiotomy cardiogenic shock (PCCS)—intraoperatively and postoperatively in the ITU. Use of ECMO preoperatively for cardiogenic shock is rare and there are no studies reporting outcomes in this population. The incidence of PCCS is estimated to be 0.5–1.5%, with the majority successfully managed with pharmacological support and use of an IABP [27]. However, a small proportion with refractory cardiac dysfunction require advanced MCS, with VA-ECMO being a commonly employed technique.

A recent multicentre study aimed to characterise the use of VA-ECMO after CABG and reported on clinical outcomes [28]. In total, 148 patients were treated with VA-ECMO for PCCS. VA-ECMO was instituted at the time of surgery for PCCS to facilitate weaning from CPB in 51.4% cases. Of the remainder, the majority of patients had a low cardiac output state, but 14.2% patients experienced cardiac arrest as the indication for VA-ECMO. Only 48.6% of patients were weaned from VA-ECMO. The in-hospital mortality was 64.2%, with survival at 1, 2 and 3 years 31.0%, 27.9% and 26.1% respectively. There was significant associated morbidity, with 23.6% of patients suffering a major neurological event, 45.3% developing renal failure requiring haemofiltration and 10.8% developing lower limb ischaemia. There were also significant bleeding complications with 51.9% of patients requiring re-exploration for mediastinal bleeding, and patients received a median of 11 units of red blood cells. It can be appreciated from this data that institution of VA-ECMO for patients’ post-CABG is associated with significant morbidity and there is poor long-term survival.

Other studies have also reported on outcomes following VA-ECMO for PCCS. Subgroup analysis of patients undergoing CABG confirms the poor survival described above. Hospital mortality ranges from 65.3 to 74% [29–31]. One study compared outcomes for patients instituted on VA-ECMO intraoperatively with those commenced in the ITU. No significant differences in survival were detected [32]. One large study demonstrated that patients undergoing isolated CABG had the best survival compared to patients undergoing other cardiac surgeries [29]. Perhaps this relates to the ability of revascularisation to contribute to myocardial recovery.

## Ventricular assist devices

Ventricular assist devices (VAD) are another advanced form of MCS which can even be used as destination therapy with patients discharged from hospital with implantable devices. There are a number of different VAD available. They can be classified by the intended duration of support (temporary vs. long-term) and by the method of implantation (percutaneous, central and implantable). When thinking about use in patients undergoing CABG, there are two situations when these devices may be used, both employing temporary devices:

- Preoperatively in patients who have cardiogenic shock, when percutaneous devices are most likely to be utilised
- Postoperatively in patients experiencing PCCS, when central devices are most likely to be utilised

### Percutaneous VAD

#### TandemHeart

The TandemHeart system (CardiacAssist) is an extracorporeal axial-flow pump. The inflow cannula is inserted percutaneously into the femoral vein, up the inferior vena cava into the right atrium and then trans-septally across into the left atrium. Blood then is pumped through a centrifugal pump which can pump up to 4 L/min, through an arterial outflow cannula into the femoral artery bypassing the LV. A continuous infusion of heparinised saline flows into the lower chamber of the pump which provides lubrication and cooling and prevents thrombus formation [33, 34]. The TandemHeart works by simultaneously contributing blood flow to the aorta, working in parallel—or ‘tandem’—to the LV. Additionally, offloading blood from the left atrium reduces LV preload, wall stress and therefore oxygen demand. This facilitates increased cardiac output and systemic perfusion pressures.

Impaired right ventricular function (to maintain left atrial volume) and severe aortic regurgitation are specific contraindications to the use of the TandemHeart. Additionally, severe peripheral vascular disease may preclude cannula placement. Patients must be anticoagulated to reduce the risk of thromboembolism. Vascular trauma and limb ischaemia are important complications of this device, as are complications associated with trans-septal puncture. If the inflow catheter is dislodged into the right atrium, a large right-to-left shunt can develop.

There is evidence that the TandemHeart is superior to IABP in providing cardiovascular support. One study examined outcomes of 117 patients with refractory cardiogenic shock despite IABP therapy in whom a TandemHeart was implanted. There were significant improvements in cardiac index, mean arterial pressure, urine output and reduction in pulmonary artery capillary wedge pressure. Despite this, the 30-day mortality was 40.2% highlighting the serious nature of the patients’ condition [35]. There have also been RCT comparing TandemHeart with IABP. Burkhoff et al. demonstrated significantly greater increases in cardiac index and mean arterial blood pressure and significantly greater decreases in pulmonary capillary wedge pressure. Overall 30-day survival and severe adverse events were not significantly different between the two groups [36]. Thiele et al. demonstrated that haemodynamic and metabolic parameters can be reversed more effectively by the TandemHeart than by standard treatment with IABP, although complications including bleeding and limb ischaemia were more prevalent [37].

#### Impella

The Impella (Abiomed) is a non-pulsatile axial-flow Archimedes screw pump designed to propel blood into the ascending aorta in series with the LV. The device is introduced retrogradely through the femoral artery (either percutaneously of via surgical cut-down) into the aorta and through the aortic valve. Blood from the LV is sucked into the inlet area near the tip of the device and is delivered into the aortic root through the outlet, thus offloading the LV. The device can provide flow rates of up to 5 L/min [34].

The Impella acts to unload the LV leading to reduced myocardial oxygen consumption, leading to improved haemodynamic status. Similar to the TandemHeart, adequate RV function is necessary to maintain LV preload. Contraindications to use of the Impella include severe aortic stenosis or presence of a mechanical aortic valve. Severe aortic regurgitation may lead to reduced efficacy. Severe peripheral vascular disease may preclude placement of the device. The commonest complications include limb ischaemia, vascular injury and bleeding requiring blood transfusion. There are also reports of significant haemolysis occurring in some patients [38].

Similarly to the TandemHeart, there are studies demonstrating superiority to IABP. One RCT demonstrated that the Impella provided superior hemodynamic support compared with standard treatment using an IABP with significant increases in cardiac index [39].

### Percutaneous VAD and CABG

#### Preoperative

In view of the findings described above, the guidelines now support the use of short-term MCS devices in patients with acute coronary syndrome-associated cardiogenic shock as a class IIa recommendation [2]. As such, there is a population of patients with recent MI and cardiogenic shock who will have devices such as the TandemHeart or Impella inserted at the time of referral for urgent or emergency CABG.

A registry analysis from the USA has examined outcomes of over 5000 patients with MI and cardiogenic shock undergoing CABG [9]. The study identified 129 patients (2.3%) who had MCS devices inserted preoperatively. The majority of these patients were bridged to surgery with an Impella. The mean LVEF of these patients was 25% and the majority of the operations were performed emergently. The operative mortality in this group of patients was 37.2% with the majority having a cardiac cause of death. This study also identified a group of patients (279, 5.1%) who required MCS intra- or postoperatively—most commonly VA-ECMO. In this group, the mean preoperative LVEF was 28%. There was a high proportion of operations being performed as ‘salvage’ (24%). The operative mortality of patients in this group was 58.4% and these patients had the highest incidence of complications compared to patients having preoperative MCS or no MCS. This data suggests that there may be some benefit to instituting MCS prior to CABG in this very high-risk group of patients.

In addition to the clinical scenario described above for cardiogenic shock, there are reports of utilising percutaneous VAD in high-risk patients in a prophylactic manner, similar to how they are increasingly being used to support patients during high-risk PCI following the PROTECT-II trial [40]. In the surgical setting, these devices have been used to support high-risk patients undergoing off-pump CABG to minimise the cardiovascular instability consequent on positioning the heart for coronary anastomoses. Several cases have been reported of successful use of the Impella and TandemHeart for this indication [41–43].

#### Intraoperative

There are limited reports of the Impella being used as a treatment option for PCCS. The largest report describes 24 patients treated in this way across three centres [44]. The authors observed that for patients who had > 1 L of residual LV ejection in addition to that offered by the Impella, the postoperative outcomes were substantially improved in comparison to similar patients managed with an IABP. For those patients with < 1-L residual LV ejection, outcomes were poor, but similar to those for patients managed with IABP. There are also recent case reports describing the use of the Impella for management of PCCS [45]. Despite these reports, other means of MCS are much more commonly employed in this setting.

### Central VAD

For patients with refractory cardiogenic shock despite IABP therapy, an alternative to VA-ECMO is the use of a central VAD to support LV function (LVAD). An LVAD comprises an inflow, a pump and an outflow. Unlike VA-ECMO, there is no oxygenator and an LVAD therefore only supports cardiac function. The inflow cannula is placed either into the left atrium or LV (differing from the strategy with VA-ECMO) and the outflow cannula placed to return blood to the ascending aorta. LVAD pump technology has evolved significantly over the last three decades. First-generation devices were pneumatic or electrical membrane pumps which generated pulsatile flow. They were bulky and noisy devices with high rates of malfunction. Now, continuous-flow centrifugal pumps are used which allows for much smaller and quieter devices. One commonly used system is the Levitronix Centrimag. The bearingless impeller-based pump has the ability to provide flows up to 10 L/min with good durability in the medium-term.

The LVAD bypasses the LV, offloading it and offers the benefits associated with reduced preload and myocardial oxygen consumption. The LV can continue to contract, but with significantly reduced work as a result of reduced preload and afterload. The cannulation strategy can protect against LV distension which can occur with VA-ECMO without an LV-venting strategy. Offloading the LV will result in a reduction of RV afterload and usually improved RV function. In patients with impaired RV function, RV failure may ensue following LVAD implantation, and this may be an indication for biventricular-VAD therapy.

The indications for LVAD therapy are well defined, and similar to VA-ECMO, patients with non-recoverable cardiac failure who are not potential transplant candidates should not be commenced on this therapy (unless it is being used as a destination therapy). LVAD can be used as a bridge to recovery in acute situations in which there is expectation of myocardial recovery—such as would potentially be the case in PCCS. It can also be used as a bridge to transplantation, and implantable LVAD is increasingly being utilised as a destination therapy for selected patients. The complications of LVAD implantation are similar to those of central VA-ECMO. Patients must similarly be therapeutically anticoagulated and thrombotic and haemorrhagic complications are the most common. Patients are similarly bed-bound with its associated complications.

### Central VAD and CABG

For patients undergoing CABG, the most likely scenario in which LVAD therapy will be considered is in the setting of PCCS. In the context of PCCS, use of LVAD has been reported in the literature, but therapy with VA-ECMO is far more commonly described. This is demonstrated by a recent multicentre study from the UK summarising salvage MCS for PCCS. The majority (85%) were managed with VA-ECMO, and only 15% had VAD implantation [46]. The largest study examining the use of LVAD use for PCCS comes from the Texas Heart Institute, describing outcomes of 22 patients in whom an LVAD was implanted either intraoperatively (*n* = 10) or in the ITU due to haemodynamic instability or cardiac arrest (*n* = 12). The overall 30-day survival rate was 41%, but it was substantially greater for those patients having LVAD implantation intraoperatively [47]. The authors conclude that timely implantation of an LVAD in the setting of PCCS avoids periods of suboptimal perfusion and resultant deleterious consequences on end-organ function and can lead to favourable clinical outcomes. Therefore, LVAD implantation can be considered as an alternative to VA-ECMO, as a means of offering MCS following CABG in selected patients.

## Predictors of poor outcome

One of the challenges that clinicians face is determining which patients developing cardiogenic shock will benefit from institution of MCS. One study has examined risk factors, which enable early identification of patients in cardiogenic shock, whose clinical condition continues to deteriorate despite maximal inotropic support and IABP support and therefore will benefit from early institution of more advanced MCS such as ECMO, Impella or TandemHeart for better end-organ preservation. Often, the window period is short and timely institution of MCS is critical for patient survival. One study performed a multivariate analysis and the risk factors identified were advanced age above 75, severely diminished mixed venous oxygen saturation and high inotropic (milrinone, noradrenaline) requirement [48].

## Conclusion

Techniques and technologies associated with MCS have evolved over the last 10–15 years and continue to improve. MCS has become commonplace and a routine part of the armamentarium in the management of patients with heart failure. Whilst a wide range of devices are now available, the potential comorbidities associated with their use remain considerable and appropriate selection is critical to the outcomes. Notwithstanding these limitations, MCS without doubt has resulted in significant improvements in the outcomes of the highest risk patients undergoing coronary revascularisation (Table 4). It is hoped that further refinements in MCS technologies in the future will continue to benefit these patients both in the short and long terms.

**Table 4:** Summary of the type of mechanical circulatory support that can be considered for each indication

Indication for MCS	Type of MCS that can be considered
Preoperatively	
Acute cardiogenic shock	IABP, Impella, TandemHeart
Symptomatic angina	IABP
Patients with critical coronary anatomy	IABP
At induction of anaesthesia	
Patients with severe ventricular impairment	IABP
Intraoperatively	
Failure to wean from cardiopulmonary bypass	IABP, VA-ECMO, Impella, LVAD
Postoperatively	
Development of low cardiac output state	IABP, VA-ECMO

*IABP* intra-aortic balloon pump, *LVAD* left ventricular assist device, *MCS* mechanical circulatory support, *VA-ECMO* veno-arterial extracorporeal membrane oxygenation
